# Main Reasons for Registration Application Refusal of Generic and Similar Pharmaceutical Drug Products by the Brazilian Health Regulatory Agency (ANVISA)

**DOI:** 10.1155/2017/7894937

**Published:** 2017-02-09

**Authors:** Ana Cerúlia Moraes do Carmo, Stefânia Schimaneski Piras, Nayrton Flávio Moura Rocha, Tais Gratieri

**Affiliations:** ^1^Office of Assessment of Synthetic Drugs Pharmaceutical Technology, General Office of Drug Products, Brazilian Health Regulatory Agency, ANVISA, Brasília, DF, Brazil; ^2^Laboratory of Food, Drugs and Cosmetics (LTMAC), School of Health Sciences, University of Brasília (UnB), Brasília, DF, Brazil

## Abstract

*Objective*. The marketing authorization of generic and similar pharmaceutical drug products involves the analysis of proposing company's administrative aspects as well as drug product technical description and scientific evaluations. This study evaluated the main reasons for registration refusal of generic and similar pharmaceutical drug products in Brazil. The aim is to help future applicants to better organize the proposal.* Methods*. A retrospective search of drug products registration processes was performed on the Brazilian Government Official Gazette from January 1, 2015, and December 31, 2015.* Results*. Drug product quality control, drug product stability study, deadline accomplishment, API quality control made by drug manufacturer, active pharmaceutical ingredient (API), and production report were the main reasons for marketing authorization application refusal of generic and similar pharmaceutical drug products in 2015.* Conclusion*. Disclosure of the reasons behind failed applications is a step forward on regulatory transparency. Sharing of experiences is essential to international regulatory authorities and organizations to improve legislation requirements for the marketing authorization of generic and similar pharmaceutical drug products.

## 1. Introduction

A generic drug is defined as a drug with the same active pharmaceutical ingredient (API), dosage form, safety, quality, and efficacy as the original proprietary drug, with which it can be interchangeable [1]. They were established in Brazil in 1999 to improve population access to low cost quality medicines. Generic drugs prices are usually at least 65% cheaper than the original ones, as they are approved after original drug patent expiration and their production do not involve molecular development and clinical studies costs [2, 3].

Similar drug products are present on Brazilian market for more years than generics. They have the same API, dosage form, strength, indication, and posology of the original proprietary drug but are identified by a brand name [1]. Only in 2003 did bioequivalence and pharmaceutical equivalence start to be mandatory for similar drugs [4]. Since 2014, all similar drugs have proved their therapeutic equivalence, so they can be interchangeable with reference drugs [5].

The Brazilian Health Regulatory Agency (ANVISA) is the national authority responsible for the minimum regulatory requirements for pharmaceutical drug products marketing authorization [6]. The review process prior registration approval is done by technical specialists based on documents submitted by an applicant. It considers company's administrative aspects and drug scientific evaluation, as established in regulatory requirements [7, 8]. For generic and similar drugs, drug scientific evaluation includes their equivalence to the brand name drug from the viewpoint of quality, efficacy, and safety, proved by bioequivalence studies, as in other agencies [7–9]. After evaluation, the registration application can be approved and questioned through major objections, when deficiencies that can be solved are identified, as other noncritical issues that require clarification, or refused, when regulatory requirements are not accomplished [1].

The rejection is a disadvantage to the applicant, who may have to review the entire process or even redevelop the product, in case the decision has reached final instances and no further appellations are allowed, to ANVISA, who spent public resources reviewing the applications, and mostly to society, which will not have access to an alternative treatment with guaranteed quality, safety, and efficacy. Moreover, scientific assessment carried on rejected applications is not published, and it prevents the study of the most important factors associated with refusal reasons. In this note, a retrospective analysis of the main reasons for marketing authorization refusal of generic and similar pharmaceutical drug products in Brazil is presented. The aim is to publish useful information for future applications, so that processes can be better prepared and time required for analysis reduced.

## 2. Material and Methods 

A retrospective search of approved and refused drug products registration processes was performed on the Government Official Gazette (GOG) from January 1, 2015, and December 31, 2015. GOG publishes brief information about process, as company name, drug product name and its API, and presentations including dosage form, strengths, container closure types, and configurations. Detailed information of each refused process was retrieved from ANVISA databank and analyzed. ANVISA databank is an internal software which contains information about drug products, that is, their applicants, application date, and motivation reports for approval or refusal.

After data review, refusal reasons were classified as administrative (nontechnical) or scientific (technical), categorized in general areas and further subdivided and detailed in specific categories, according to subjects described on particular regulatory regulations.

## 3. Results 

Between January 1, 2015, and December 31, 2015, 272 new, generics, and similar pharmaceutical drug products applications were published on Government Official Gazette. From this total, 136 products were approved: 25 new drugs (10%), 99 generics (36%), and 12 similar drugs (4%). 136 generics and similar products had their registration refused by ANVISA. Any new drug product was refused. Six of them referred to clone petitions, simplified application linked to a matrix petition, which contains all technical and clinical information requested for drug product registration. Clone is based on a matrix petition and they differ exclusively in drug product name, packaging layout, and legal information [10]. Hence, 130 refused reports from 55 different applicants were considered in this note: 93 (35%) generic drugs and 37 (14%) similar drugs.

From 130 refused reports, 62 (47%) refused reports are related to drug products produced in Brazil; 26 (21%) are drug products from other countries: India (17; 13%), Chile (3; 2%), Argentina (1; 1%), Slovenia (1; 1%), Uruguay (1; 1%), Spain (1; 1%), Germany (1; 1%), and Turkey (1; 1%). 42 (32%) reports did not inform where drug product was produced.

Approved new drug products corresponded to 10% (25) of registered products. 5% (13) of Brazilian new products have been already registered by both FDA and EMA, 1% (2) only by FDA, and 1% (2) only by EMA [11, 12].

**Figure 1:**
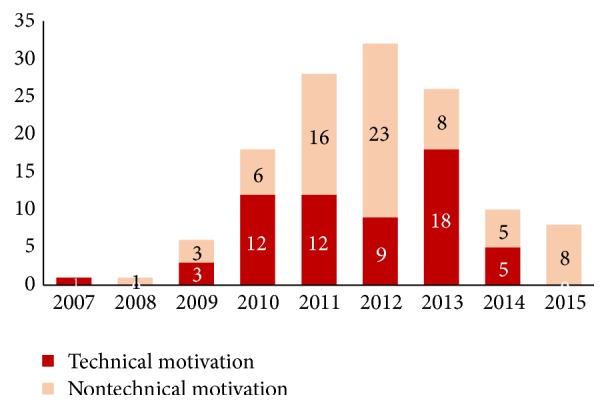
Number of refused marketing authorization applications in accordance with process submission year.

Retrieved processes were submitted between 2007 and 2015. Major nontechnical refusals occurred on documents applied on 2011 and 2012 (Figure 1).

The retrospective analysis revealed 501 reasons for refusal. Each reason was classified in general areas according to major themes described in registration regulations and detailed in specific categories [7, 8] (Table 1). 21 reports presented just one reason for refusal.

Technical reasons corresponded to 84% (420) of refused registrations. Nontechnical reasons (16%; 81) included deadline accomplishment, preliminary analysis, and documentation.

Drug product quality control was the main reason for registration refusal. Analytical method validation problems, related to quality control, involved lack of specificity, linearity, accuracy in assay, dissolution, and impurity methods.

Main reasons related to API quality control were also analytical validation problems including absence of validated analytical method for impurities assay and lack of specificity in impurities analytical method.

**Figure 2:**
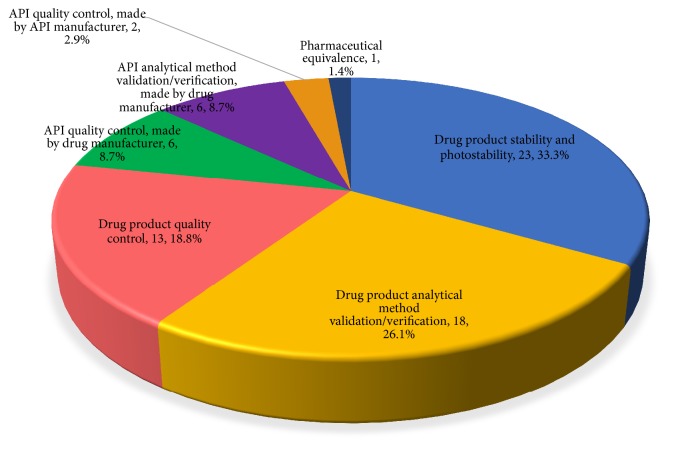
Distribution of problems related to impurity which led to refusal of marketing authorization application for generic and similar pharmaceutical products.

The topic related to impurities represented an important registration reason for refusal, which was related not only to drug product quality control but also to drug analytical method validation or verification, drug stability and photostability, and API quality control performed by drug product manufacturer and API manufacturer, as represented in Figure 2.

## 4. Discussion

In the analyzed period, refused registrations corresponded to 50% of all published marketing authorization processes. This corresponds to a large quantity when compared to US Food and Drug Administration (FDA), which refused 12% applications in 2009; 18% in 2010; 15.5% in 2011; 9.4% in 2012 [13]. Between 2000 and 2012, 80 new drug products were not approved in the USA: 76 (95.0%) due to safety, efficacy, or both deficiencies and 4 (5.0%) due to quality [14]. In Europe, in 2009, 19 (40%) of new drug products received a negative opinion of European Medicines Agency (EMA) or were withdrawn by the applicant prior to receipt EMA opinion [15].

Pignatti et al. (2002) have researched issues raised during the review of drug applications submitted for approval to the EMA and their impacts on outcomes. 32 of the 111 applications reviewed between September 1997 and April 2000 (29%) were rejected (29 applications were withdrawn, and 3 received a negative opinion). Quality major objections were related to API quality control (14.4%), pharmaceutical development of the finished product (12.6%), stability of the finished product (10.8%), finished product quality control (9.0%), characterization of API on generics (8.1%), biological development (8.1%), and stability of the active substance (6.3%) [16].

In Europe, major objections are sent to the applicant during the review. Applicants have to accomplish them in a predetermined period of time [16]. The process is essentially the same in Brazil [17]. Failure to solve all major objections may lead to a refusal. Faced with this probability, applicants often prefer to withdraw their application in Europe (almost 100% of refused marketing authorization was due to being withdrawn). The same does not happen in Brazil. All refused registration received a negative opinion from ANVISA. There is also an instrument to being withdrawn [18] but it is not as representative.

Particularly the large number of nontechnical refusals (16%) was not expected, since regulations orienting the submission process were already available. Application submission procedure was established in 2005 [17], detailed in 2012 [19] and 2013 [20] through orientations. Therefore, a possible explanation for the persistency of nontechnical refusals would be that although regulatory requirement was available, it was not applied as they should be, neither by companies nor by ANVISA. The procedure began to be better applied only after both orientations [19, 20]. A great majority of processes applied on 2011 and 2012 refused due to nontechnical motivation (80%) reinforce this hypothesis. In 2012, a preliminary analysis procedure was determined, by which a screening approach should briefly review all applications received, checking if it was sufficiently complete to permit a substantial review. Orientations for helping process preparations and correct submission are one of ANVISA's responsibility. Hence, transparency actions as this one are strongly encouraged, so new processes are submitted in accordance with guidelines.

FDA also has established some requirements to refuse submissions that are not sufficiently complete to permit substantive review [21]. Repairing an incomplete file during the analysis is a waste of resources, because it needs many cycles of FDA response and applicant repair [13].

Apart from this, deadline accomplishment, a nontechnical reason, was the third major reason of refusal, mainly due to absence of clone drug product petition. Clone drug product is identical to another one, named matrix petition, differing exclusively in name and labeling [10]. It was a particular situation in the year 2015, due to deadline established by regulation to clone adequacy. It will not happen in the next years [10].

Data content of quality, safety, and efficacy to be presented to ANVISA (Table 2) are very similar to that required by Common Technical Documentation (CTD) established by ICH and World Health Organization [22–26]. Hence, ANVISA regulations are being developed in consonance with international regulatory authorities. However, as Brazilian drug producers are still responsible for 74% of all refused processes and no Brazilian drug product was approved to American or European market on 2015, it can be concluded that they are still not able to follow Brazilian regulation or foreign ones [12, 27]. This data reinforces the importance of transparency initiatives to ameliorate technical quality of national drug products and expand internal and international generic and similar drugs market.

Drug product quality control problems had the highest occurrence (13.3%). For pharmaceutical drug products registration, quality, safety, and efficacy for the proposed use have to be recognized through scientific evidence and analysis [1] using established methods accepted by ANVISA [28]. When a specific analytical method is not described in any official pharmacopeia accepted by ANVISA, it has to be validated [7, 8, 29]. In USA, the concept is similar [30]. The Brazilian current regulation on validation of analytical procedures is Resolution RE number 899/2003, which revoked the first one, Resolution RE number 475 (March 19, 2002) [29, 31]. Since then, there are regulatory requirements for this theme which are not yet fully accomplished. Brazilian regulation is also quite similar with ICH guideline. The main difference is robustness requirement and precision parameter relative standard deviation [32]. However, such differences do not justify the large number of rejections on this subject. Regulatory requirements establish which parameters must be validated, but they do not bring detailed descriptions of how to perform the assays. We believe such absence may be the main reasons to stand validation as one of the most common reasons for refusal. The validation procedure is an important step of method development [33] and it has to be suitable for each product [32]. Main challenge for a method validation may not be the method development itself rather the experiments planning and results interpretation based on proper statistical analysis. An iconic representation of this may be found in one of the process submitted in 2012 in which specificity was not proved for both assay and impurity test. The regulatory requirement states that specificity should be conducted during the validation of identification tests for the determination of impurities and the analyte [29]. For the analyte assay and impurity test, specificity can be determined comparing the results obtained on samples spiked with appropriate levels of impurities or excipients, with unspiked samples, to demonstrate that the results are unaffected by the presence of these materials. When a possible impurity is not available, it is necessary to perform forced degradation tests [29]. In the example depicted here, impurities standards were not available, and the absence of forced degradation test was not justified, demonstrating lack of proper experiment planning and noncompliance with regulatory requirement, consequently. Hence, the necessity of training programs is evident [33].

Drug product stability study was the second main reason for marketing authorization refusal. It is an obligatory study for drug product registration [7, 8]. Stability first regulation from 1996 had already discussed the importance of tests to effectively assess the dosage form, including assay, impurity, and dissolution. In addition, it established that evaluation methods should be validated and stability-indicating [34]. Nowadays, Resolution RE number 01/2005 is the current regulation [35]. It brings some obligatory tests or the possibility for justifying its absence. Nonetheless, results demonstrated that despite the increased rigidity of the current legislation, it has been published for more than 10 years, so it is still lagging when compared to the Stability ICH guidelines [36]. Even so, some refusals motivations were already covered in the Brazilian legislation for 30 years. Moreover, many stability reasons are also related to analytical method, validation and development problems, as already discussed. Stability specifications have to be determined based on each drug product. Dissolution methods and specification recommended by other regulatory agencies to methods not described in official compendia, as FDA-Recommended Dissolution Methods [37], cannot be applied without prior critical analysis, because they are not always suitable for Brazilian products. They are just aids of industry personnel development.

API quality control, made by drug product manufacturer, is fourth main refusal reasons, after deadline accomplishment, already discussed. Considering that the API quality is critical to drug product quality, it is essential that drug manufacturers assure and confirm that API accomplishes quality requirements. It means drug products manufacturers have to establish internal API quality control specifications and methodology. Although this information is present in registration regulatory requirements, absence of API methodology development is still a failure point on drug registration. Internal API quality control specifications and methodology should be adopted based on impurity profile and residual solvents arising from API manufacturer synthetic route, official compendia, and also international guidelines as ICH quality guidelines [36]. Although international guidelines do not bind ANVISA decisions, it serves as source and guidance.

Two items related to API manufacturers are important refusal reasons: API and API stability studies. When a drug product is approved, an API manufacturer is also approved for that drug [7, 8]. Therefore, API manufacturer has to comply with Brazilian regulatory requirements. Within API manufacturer category, great part of denials corresponded to lack of stability studies performed on Brazilian climatic zone. Brazil is classified as IVb climatic zone [38]. It is mandatory to present complete accelerated stability studies and at least long term stability studies protocol in IVb climatic zone [39, 40]. It is a controversial point of evaluation, and ANVISA has received several complaints by industry. The problem is that many international API manufacturers refuse to comply with Brazilian regulation, as countries representing major consumer markets belong to climatic zones of milder conditions. Therefore, they usually do not perform such studies on the Brazilian stipulated condition.

Equally important reason for process refusal was the absence of complete production report, which allows the evaluation of current production process and the possibility of future postapproval changes. Production reports can reflect absence of GMP accomplishments and disconnection between production, development, and regulatory departments in pharmaceutical industry. The absence of complete production reports may be indicative that these departments did not work together during product development and regulatory application preparation.

Production reports were followed by pharmaceutical equivalence. Main problem related to pharmaceutical equivalence was related to analytical method validation: the method was not validated or did not comply with validation regulatory requirement [29]. If it happens, pharmaceutical equivalence and interchangeability cannot be proved. Pharmaceutical equivalence is the basic principle of generic and similar drugs. It must be performed by specialized centers to avoid losing all investments and all drug product studies [41]. The same happens with bioequivalence studies and other pillars to prove efficacy and safety of generic and similar drugs. If study is reproved or not presented, drug product will not be able to get marketing approval.

Other reasons for marketing authorization denial included inadequacies on dissolution test. Dissolution method has to be rugged and reproducible for routine operation and capable of being transferred [42] and discriminative, which means to be sensitive to formulation and process variables that can affect dissolution rate [41] and, in some cases, to reproduce biopharmaceutical product performance [43]. The most common way to challenge discriminatory power of a method is to test formulations with changes in critical process parameters and it is completely dependent on the formulation [43]. Thus, even there is a dissolution method described in a pharmacopeia, it is necessary to check if it is suitable and discriminative for that drug product [44], differently of what is regulated [17, 19, 20, 41]. As this information is not clear in regulation, it can justify this refusal reason.

Reasons for registration denials regarding impurities, if considered as a separate subject, would represent the third most frequent cause for refusal, with 13.7% of citations. Impurities are a universal test for both API and drug products [45]. A drug product has to have recognized quality, identity, activity, quality, purity, and safety and no raw material can be used in drug manufacturing without having been checked for acceptable quality [1]. Since 1976 evidences of the importance of API and drug product purity are described [1]. In 2008, Technical Report IT number 01 (July 15, 2008) was disclosed, reaffirming the importance of impurities identification and quantification on stability and analytical methods validation [31, 35, 46]. IT number 01/2008 suggested conditions for forced degradation studies to comply with current legislation and introduced limits of impurities qualification, identification, and notification, which were regulated only in 2013 [47, 48].

Impurities items were mainly related to noncompliance with stability studies and analytical methods validation regulations, as specific resolution only comes into effect from the end of 2015 for new registration requests [48]. Developing specific methods for identification, quantification, and, in many cases, impurities qualification is probably what generated such a refusal index.

Compared to other regulatory agencies, ANVISA can be considered as a new one. Technical standards are still being developed and improved, in consonance with international agencies in many cases. Topics as drug stability, analytical method validation, and impurities identification are being continuously discussed in the agency [49]. Reported data shows that ANVISA legislation has advanced on technical requirements, but companies still violate basic aspects, which are nationally regulated for at least a decade and widely discussed scientifically. The high proportion of applications rejected highlights a gap between regulatory expectations and applicants development and submission strategies. To solve these problems, transparency is already a concern of international regulatory agencies. Detailed information about approved drugs, serious adverse drug reactions, and other pharmacovigilance relevant data have been standardized [50, 51]. Brazil incorporates the essence of transparency [52], but information provision is mostly by request. Proactive disclosure is still a challenge. Undoubtedly, established standards from international health regulatory agencies could be a reference to openness and disclosure approaches in this area [53].

## 5. Conclusion

Disclosure of the reasons behind failed applications is a step forward on regulatory transparency, which can be useful for both industry and ANVISA to ameliorate marketing authorization process. Drug product quality control, drug product stability study, deadline accomplishment, API quality control made by drug manufacturer, and API and production report were the main reasons for registration application refusal of generic and similar pharmaceutical drug products in 2015. Hence, producers of generic and similar pharmaceutical drug products are encouraged to allocate resources and training on these main issues so they can properly ensure technical quality of developed products and a successful marketing authorization.

## Figures and Tables

**Table 1:** Distribution of main reasons for refusal to approve generic and similar pharmaceutical drug products.

Classification (general area and specific categories)	Quantity (*n*)	%
*Drug product quality control *	*67*	*13.3%*
Analytical method validation	26	5.2%
Absence of impurity control	7	1.4%
Absence of obligatory tests	6	1.2%
Absence of adequate justification for proposed limits for impurities	5	1.0%
Partial analytical method validation	5	1.0%
Reproved method or specification	4	0.8%
Wrong calculation	3	0.6%
Others	11	2.2%

*Drug product stability study *	*62*	*12.4%*
Absence of impurity control	17	3.4%
Refusal due to reproved quality control	8	1.6%
Nonstability indicating assay method	7	1.4%
Incomplete study	5	1.0%
Absence of obligatory quality control tests	4	0.8%
Disagreement between dissolution specification and results	4	0.8%
Absence of reconstitution stability study	3	0.6%
Nonspecific method for degradation products	2	0.4%
Others	12	2.4%

*Deadline accomplishment*	*50*	*9.9%*
Absence of clone drug product petition	33	6.6%
Nonaccomplishment of objections answer deadline	13	2.6%
Others	4	0.8%

*API quality control, by drug product manufacturer*	*41*	*8.2%*
Analytical validation problems	13	2.6%
Absence of method or analysis of residual solvents	7	1.4%
Absence of method or analysis of impurities	6	1.2%
Absence of obligatory tests	4	0.8%
In disagreement with compendial standard	3	0.6%
Absence of certificate of analysis	2	0.4%
Others	6	1.2%

*Active pharmaceutical ingredient (API)*	*41*	*8.2%*
API quality control faults	25	5.0%
Lack of API polymorphic form proof	5	1.0%
Lack of synthesis route	3	0.6%
Nonaccomplishment of objections answer deadline	2	0.4%
Absence of documents	2	0.4%
Others	4	0.8%

*Production report *	*29*	*5.8%*
Production report did not include all stages of production process	8	1.6%
Production process was nonreproducible	6	1.2%
GMP noncompliance	5	1.0%
Different batch sizes	3	0.6%
Generic formulation with different API from the reference drug	2	0.4%
Others	5	1.0%

*Pharmaceutical equivalence *	*27*	*5.4%*
Analytical method validation	10	2.0%
Reference drug product with unapproved efficacy and safety	4	0.8%
Reproved quality control method	3	0.6%
Reproved	4	0.8%
Others	6	1.2%

*API stability studies *	*23*	*4.6%*
Lack of stability studies on Brazilian climatic zone	16	3.2%
Nonstability indicating methods	4	0.8%
Absence of accelerated stability study	2	0.4%
Others	1	0.2%

*Dissolution*	*23*	*4.6%*
Nondiscriminative methods	10	2.0%
Inadequate dissolution quality control specification	4	0.8%
Others	9	1.8%

*Bioequivalence studies *	*17*	*3.4%*
Reproved	10	2.0%
Absent	5	1.0%
Studies done with drug products that are no longer considered reference	2	0.4%

*Excipients quality control*	*16*	*3.2%*

*Preliminary analysis*	*16*	*3.2%*

*Documentation*	*15*	*3.0%*
Absence of current GMP certificate	11	2.2%
Others	4	0.8%

*Drug product photostability*	*13*	*2.6%*
Absent	7	1.4%
Absence of degradation products control	5	1.0%
Others	1	0.2%

*Minor reasons *	*61*	*12.7%*

*Total*	*501*	*100.0%*

**Table 2:** Obligations of an application.

Area [6, 7]	Obligations [6, 7]	Comment
Administrative documentation	(i) Payment of health surveillance fee (ii) GMP certification	The absence of fee payment, operating authorization, and GMP certification lead to marketing authorization refusal without substantive review [12]
	(iii) Operating authorization	A special operating authorization is necessary when controlled drugs are manipulated
	(iv) Sanitary permit	
	(v) Petition forms	
	(vi) Certificate of technical responsibility	
	*For imported drug products*	For imported drug products, the absence of CPP and GMP certification causes marketing authorization refusal without substantive review [12]
	(i) Certificate of Pharmaceutical Product (CPP)	
	(ii) GMP certification	
	(iii) Importer quality control specifications	

Production report	(i) Batch master record	Production report should be the standard one for production of either pilot or industrial batches
	(ii) Production process and equipment	
	(iii) Industrial batch size	
	(iv) Three pilot batches' record copies	Pilot batches records must have the same processes established on batch master record

Active pharmaceutical ingredient (API)	(i) Synthetic route	Describe starting material, solvents, and intermediates
	(ii) Analytical methods and specifications adopted	(i) Both API and drug product manufacturers have to present analytical method and specifications
		(ii) When a specific analytical method is not described in any official pharmacopeia accepted by ANVISA, it has to be validated [6, 7]
	(iii) API certificate of analysis	API certificate of analysis also has to be presented. Drug product manufacturer has to apply API certificates from the API batches used on drug product pilot batches [6, 7]
	(iv) Mainly impurities	Main impurities have to be monitored on quality control tests
	(v) Chirality data	Chiral forms may have different pharmacological effects. They are related to efficacy and safety of a drug product
	(vi) Polymorphism data	Polymorphism can affect solubility and dissolution rate of a drug product. It directly impacts bioavailability. API polymorphic forms have to be monitored until the expiration date, using proper analytical methods for physical characterization
	(vii) Stability and photostability studies	Stability studies must be performed in Brazilian climatic zone, IVb: 30°C ± 2°C; 75% ± 5%

Drug product quality control	(i) Analytical methods and specifications adopted	When a specific analytical method is not described in any official pharmacopeia accepted by ANVISA, it has to be validated [6, 7]
	(ii) Certificate of analysis of three pilot batches	(i) Methods must be specific. To prove specificity, forced degradation studies must be performed with the drug product
		(ii) Forced degradation studies are also important for prediction of possible compounds generated on stability studies, production process, and interaction between excipients and API

Drug product stability study	Stability study report from three pilot batches	(i) Stability indicating methods must be used to determine, with accuracy, the content of the drug product, degradation products, and other components, without any interfering species
		(ii) Methods must be validated
		(iii) Specification should be stablished according to drug product on analysis

Therapeutical equivalence	(i) Pharmaceutical equivalence	Pharmaceutical equivalence must compare drug product biobatch and reference drug product. Biobatch consists of the drug product batch used on bioequivalence studies
	(ii) Bioequivalence	Bioequivalence is an in vivo obligatory study which compares the bioavailability of a reference drug product and generic or similar drugs
